# Individual Differences in Infant Oculomotor Behavior During the Viewing of Complex Naturalistic Scenes

**DOI:** 10.1111/infa.12049

**Published:** 2014-03-28

**Authors:** Sam V Wass, Tim J Smith

**Affiliations:** MRC Cognition and Brain Sciences Unit; School of Psychological SciencesBirkbeck College, University of London

## Abstract

Little research hitherto has examined how individual differences in attention, as assessed using standard experimental paradigms, relate to individual differences in how attention is spontaneously allocated in more naturalistic contexts. Here, we analyzed the time intervals between refoveating eye movements (fixation durations) while typically developing 11-month-old infants viewed a 90-min battery ranging from complex dynamic to noncomplex static materials. The same infants also completed experimental assessments of cognitive control, psychomotor reaction times (RT), processing speed (indexed via peak look during habituation), and arousal (indexed via tonic pupil size). High test–retest reliability was found for fixation duration, across testing sessions and across types of viewing material. Increased cognitive control and increased arousal were associated with reduced variability in fixation duration. For fixations to dynamic stimuli, in which a large proportion of saccades may be exogenously cued, we found that psychomotor RT measures were most predictive of mean fixation duration; for fixations to static stimuli, in contrast, in which there is less exogenous attentional capture, we found that psychomotor RT did not predict performance, but that measures of cognitive control and arousal did. The implications of these findings for understanding the development of attentional control in naturalistic settings are discussed.

## Introduction

Previous research has used standardized experimental assessments to study how individual differences in attention manifest during infancy (Colombo & Mitchell, 2009; Courage, Reynolds, & Richards, 2006; DiLalla et al., 1990; Rose, Feldman, & Jankowski, 2002, 2009; Rose, Feldman, Jankowski, & Van Rossem, 2011a; Rose, Feldman, & Jankowski, 2012). Comparatively little research, however, has studied how individual differences on these experimental assessments relate to individual differences in how attention is spontaneously allocated in naturalistic settings. This leaves a number of important questions unanswered. For example, individual differences in attention during infancy, as assessed using habituation and reaction time (RT) paradigms, have been shown to relate to long-term outcomes on language and executive function measures (Rose et al., 2009, 2012)— but are these relationships observed because early attentional control leads to better (more efficient) orienting and learning behaviors in naturalistic contexts (see e.g., Samuelson, Smith, Perry, & Spencer, 2011; Yu & Smith, 2011a,b)? Or are they observed because these experimental assessments tap some underlying “pure” aspect of cognition that is independent of naturalistic orienting? This question, which is relatively under-addressed in the literature (although see Aslin, 2009; de Barbaro, Chiba, & Deak, 2011; Hunnius, 2007; Rose, Feldman, & Jankowski, 2011b; Smith & Sheya, 2012), is the focus of the present article.

Naturalistic behaviors can be studied at different spatio-temporal scales (Hutchins, 1995). The present article concerns the microdynamics of spontaneous attention, namely the duration of fixations (typically in the order of hundreds of milliseconds) during unconstrained orienting to naturalistic scenes.

### Eye movements during unconstrained orienting

The central part of the retina is the fovea, which has a diameter of about 1 millimeter in infants; it comprises <1% of the retina but takes up over 50% of the visual cortex (Yuodelis & Hendrickson, 1986). When viewing a visual array, we spontaneously manifest a sequence of eye movements to ensure that light from objects of interest is projected onto the fovea (Holmqvist et al., 2011; Land & Tatler, 2009). Our eyes alternate between periods in which the eye is static and visual processing occurs (fixations), and rapid eye movements (saccades) during which visual processing is suppressed (Matin, 1974). Saccades, which separate fixations, can span as little as 0.1° of visual angle; fixations (in adults) generally last 200–500 msec (Holmqvist et al., 2011).

#### Fixation durations in adults

Within the adult literature, a body of research exists using fixation duration as an index of online cognitive processing demands (Henderson, 2003; Henderson & Pierce, 2008; Henderson, Chanceaux, & Smith, 2009; Henderson & Smith, 2009; Nuthmann, Smith, Engbert, & Henderson, 2010; Rayner, 1998; Smith & Henderson, 2009). Research has suggested that bottom-up visual features such as edges and motion (Itti & Koch, 2001), luminance (Loftus, 1985) or clutter (Henderson et al., 2009) can influence fixation duration, as well as top-down factors such as viewing task and personal preference (Rayner, 1998; Tatler & Vincent, 2008; Yarbus, 1967). Fixations are longer for semantically inconsistent objects such as an octopus in a farm (Loftus & Mackworth, 1978; but see Henderson & Hollingworth, 1998). These findings point to the utility of fixation duration as an index of online cognitive processing demands that varies as a function of the information at the point fixated.

Additionally, however, other studies with adults have suggested that individual differences in fixation duration appear stable across different types of visual stimuli. For example, significant relationships have been demonstrated between fixation durations during the viewing of line drawings, photographs, computer-rendered scenes, and faces (Castelhano & Henderson, 2007; see also Andrews & Coppola, 1999). Similar findings have been reported in chimpanzees (Kano & Tomonaga, 2011a,b).

Research with animals and adults has suggested that a number of components may influence fixation duration (e.g., Findlay & Walker, 1999a,b; Henderson, 2013; Nuthmann et al., 2010). The first component is thought to be the oculomotor command to move to a peripheral target based on saliency computations originating in the visual cortex and implemented via brainstem circuitry, including the superior colliculus (Becker & Jürgens, 1979; Findlay & Walker, 1999a,b). The second is the requirement of processing the visual information that is present at the point fixated (Engbert, Longtin, & Kliegl, 2002; Engbert, Nuthmann, Richter, & Kliegl, 2005; Nuthmann et al., 2010; Rayner, 1998). This is thought to be implemented via online inhibitory control of eye movements exerted via the frontal eye fields, the superior colliculus, and substantia nigra (Findlay & Walker, 1999a,b). In addition, work using scene onset delay paradigms has suggested that some fixational eye movements are triggered irrespective of information at the point fixated, suggesting that internal saccade timer mechanisms also play a role in guiding eye movement behavior (Henderson & Pierce, 2008; Henderson & Smith, 2009; Morrison, 1984; see also McAuley, Rothwell, & Marsden, 1999). Individual differences in each of these three components may separately influence fixation duration and have been successfully modeled in adults during simple psychomotor tasks (Carpenter & Williams, 1995), reading (Engbert et al., 2002, 2005; Reichle, Rayner, & Pollatsek, 2003), and scene viewing (Nuthmann et al., 2010).

Another area of research has looked at within-participant variance in fixation duration. This may relate to the role that internal saccade timer mechanisms play in influencing fixation duration relative to other, endogenous factors (Aston-Jones, Iba, Clayton, Rajkowski, & Cohen, 2007; de Barbaro et al., 2011). For example, research has suggested that adults with autism spectrum disorders (ASD) show reduced modulation of fixation duration by the semantic content at the point fixated (congruous versus incongruous) (Benson, Castelhano, Au-Yeung, & Rayner, 2012; see also Benson, Piper, & Fletcher-Watson, 2009; Kemner, Verbaten, Cuperus, Camfferman, & van Engeland, 1998; Landry & Bryson, 2004). “Inflexible” orienting styles, in which saccades are relatively more driven by internally generated saccade timing mechanisms, have been contrasted with “flexible” orienting styles in which process monitoring and the viewer's interest in the point fixated play a relatively greater role in determining fixation duration (Henderson & Smith, 2009; Nuthmann et al., 2010; see also Aston-Jones, Rajkowski, & Cohen, 1999; de Barbaro et al., 2011). This suggests the importance of studying variance in fixation duration as a parameter of individual differences independent of mean fixation duration.

#### Fixation durations in infants

An infant's visual processing system differs from an adult's in a number of ways (Bronson, 1974; Colombo, 2001; Colombo & Cheatham, 2006; Hunnius, Geuze, & van Geert, 2006; Hunnius, 2007; Johnson, 1991, 1995, 2010; Johnson, Posner, & Rothbart, 1991; Schiller, 1985).^1^ A number of detailed investigations of spontaneous fixational eye movement patterns in infants came from Bronson, who used a 9 Hz corneal reflection eyetracker to hand-code eye movements while viewing static, geometric shapes (Bronson, 1990, 1991, 1994; although see also Aslin & Salapatek, 1975; Haith, Bergman, & Moore, 1977; Leahy, 1976; Salapatek & Kessen, 1966, 1973 for earlier work in this field). Bronson observed that when 1- to 2-month-old infants view static visual stimuli, they show a series of long fixations that are close together (Bronson, 1990); by 3–4 months, a more controlled scanning method has emerged for static stimuli, with a greater proportion of shorter (<500 msec) fixations (Bronson, 1994). When the stimulus was flickering, however, the infants' scanning characteristics reverted to those found at younger ages (see also Atkinson, 2000; Bronson, 1990; Hood & Atkinson, 1993). These developmental changes have been widely attributed to the maturation of volitional eye movement control from the frontal eye fields, exerted via the substantia nigra and the superior colliculus, although this attribution remains speculative (Bronson, 1994; Hunnius, 2007; Johnson, 1991, 2009; Schiller, 1985).

Some work has noted individual differences in spontaneous fixational eye movement patterns during infancy. Bronson (1994) measured how individual differences in visual attentiveness change during the course of testing in typically developing (TD) 6- to 13-week-old infants; he found that decreases in visual attentiveness were associated with more time spent in prolonged fixations during the viewing of static, geometric shapes. Visual attentiveness was measured from an index derived from breaks in contact with the eyetracker and the difference in pupil size between trials. de Barbaro et al. (2011) played short musical clips between six TV monitors positioned in a 360° circle around TD 6- to 7-month-old infants and coded fixation durations as well as latencies to reorient visual attention, the percentage of trials in which an infant fixated the target monitor, and the proportion of time spent looking at “target” versus “nontarget” areas. They found that fixation durations, total looking time to the target areas, and likelihood to reorient toward targets all mapped onto a single factor, which they named a “vigilance factor” based on similar findings from the animal literature (Aston-Jones et al., 1999; Aston-Jones & Cohen, 2005).

### The current study

**Figure 1:**
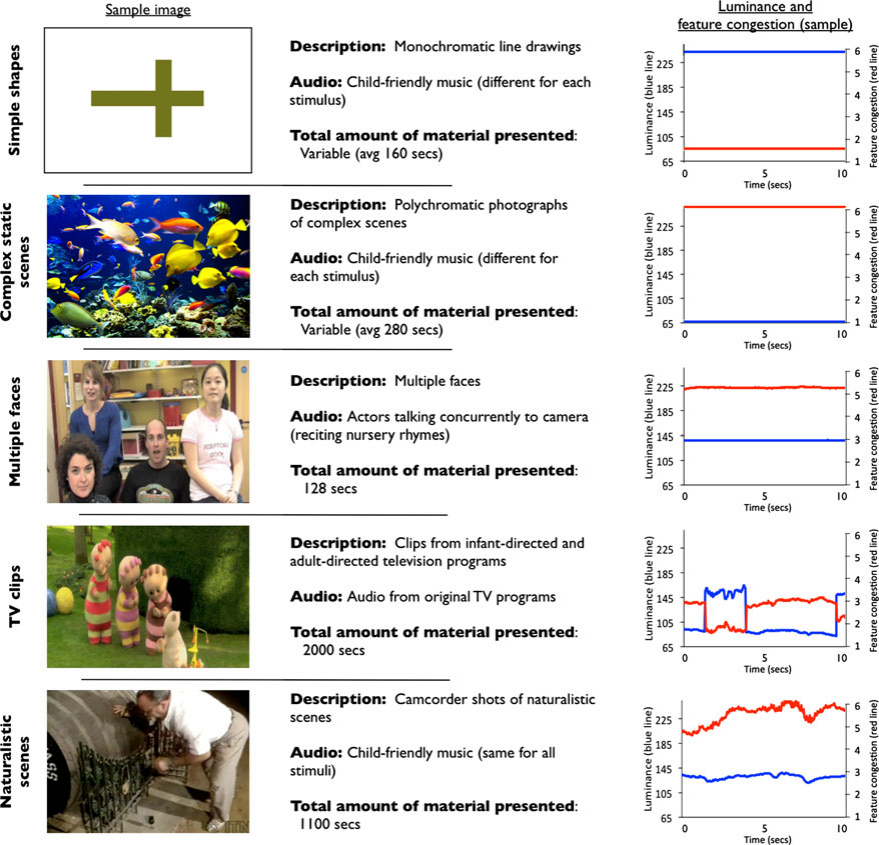
Details of the viewing material presented. The first column shows a sample image from each category. The third column shows the luminance and feature congestion. For the dynamic images, this has been calculated frame by frame. Luminance was calculated in Matlab using the 1976 CIE L*a*b* (CIELAB) color space. Feature congestion was calculated using processing scripts written by Rosenholtz et al. (2007).

We wished to examine how individual differences in attention, as assessed using standard experimental paradigms, relate to individual differences in how attention is spontaneously allocated in naturalistic contexts. To address this, we recorded spontaneous eye movement patterns while TD 11-month-olds viewed a 90-min battery of mixed viewing materials over five laboratory visits. Previous research points to differences in viewing behavior contingent on the nature of the viewing material presented, static versus dynamic, with dynamic stimuli exhibiting significantly longer duration fixations due to extra foveal visual activity (Dorr, Martinetz, Gegenfurtner, & Barth, 2010; Smith & Mital, 2013). We elected therefore to present a range of viewing materials: noncomplex static (monochromatic line drawings of simple geometric shapes), complex static (detailed photographs, for example, of fish in a fish tank), multiple dynamic faces (a steady camera shot of four actors talking concurrently to the screen), infant- and adult-directed TV clips, and videos of naturalistic scenes (e.g., short movies of everyday point-of-view scenes of restaurants, indoor scenes, and so on (see Figure 1)). The same infants also completed a battery of widely used experimental assessments of attention. Eleven-month-old infants were chosen because this age is often described as a “transitional” age associated with the first emergence of endogenous attentional control (Colombo & Cheatham, 2006; Courage et al., 2006). We judged that selecting this age range, as opposed, for example, to younger infants, might increase the range of relationships observed in this initial study.

Our first research question was, can stable individual differences be identified in the frequency of spontaneous eye movements during unconstrained orienting in infancy? We hypothesized that we would identify stable individual differences in fixation durations during unconstrained viewing, based on similar findings from the adult (e.g., Castelhano & Henderson, 2007) and primate (Kano & Tomonaga, 2011a) literature.

Our second question was, what are the factors that drive individual differences in the microdynamics of naturalistic attention? To assess this, we administered five different tasks that are thought to assess cognitive control, psychomotor RTs, processing speed (assessed via peak look during habituation), and arousal (indexed via tonic pupil size). We examined how interparticipant differences on each of these tasks relate to differences in (1) mean fixation duration for dynamic and for static visual stimuli, and (2) intraparticipant variance in fixation duration (separately for dynamic and static stimuli).

The following experimental tasks were used:

1. *Psychomotor RTs – noncompetition*. This measures the latency to reorient visual attention between two spatially discrete targets presented consecutively (Elsabbagh et al., 2008; see also DiLalla et al., 1990). It is thought to index low-level oculomotor control networks (Karatekin, 2007). Using different but comparable measures, de Barbaro and colleagues observed a medium correlation between attentional reorientation latencies and spontaneous fixation durations during the viewing of TV clips in TD 6- to 7-month-old infants (de Barbaro et al., 2011). We predicted therefore that we would find a positive relationship between psychomotor RTs and fixation duration—that is, faster RTs associated with shorter fixation durations.
2. *Psychomotor RTs – disengagement*. This is derived by comparing RTs between two conditions: noncompetition (target 1 disappears as target 2 appears, as in task 1) and competition (target 1 remains on-screen as target 2 appears). Attentional disengagement latencies are calculated as the difference between RTs in the competition and noncompetition conditions—that is, the “cost” of the presence of the central target (CT) (Elsabbagh et al., 2008). Attentional disengagement is thought to require additional cortical involvement from prefrontal and parietal areas (Csibra, Johnson, & Tucker, 1997; Csibra, Tucker, & Johnson, 1998). Hunnius et al. (2006) recorded disengagement latencies (using a substantially different paradigm) and fixation durations to dynamic stimuli longitudinally from 10 TD infants between the ages of 4 and 26 weeks and found no evidence of a positive association between the development of fixation durations and disengagement on the level of the individual infant; however, only a small amount of viewing material was collected (30 sec per infant). Frick, Colombo, and Saxon (1999) identified relationships between disengagement latencies and look duration in 3- and 4-month-old infants, and Kano and Tomonaga (2011a) identified significant correlations between disengagement latencies and fixation durations in chimpanzees. Despite these inconsistent findings we predicted that we would find a positive relationship between attentional disengagement latencies and fixation duration—that is, faster RTs associated with shorter fixation durations.
3. *Processing speed (indexed via peak look duration during habituation)*. Static stimuli are presented repeatedly across discrete but contiguous trails until the infant is judged to have habituated; infants' peak look (i.e., the duration of the longest unbroken look) has been reported as this is thought to be the primary factor that drives both individual and developmental differences in visual habituation during infancy (Colombo et al., 2004; Colombo & Mitchell, 1990; Colombo & Mitchell, 2009). Peak look during habituation has been described as an index of processing speed (Colombo & Mitchell, 2009; Rose et al., 2002). To our knowledge, no research has yet assessed the relationship of this measure to fixation durations during spontaneous orienting. However, based on the relationships that have been hypothesized between processing speed and peak look during habituation, and between processing speed and fixation duration (Nuthmann et al., 2010), we predicted that we would find a positive relationship between peak look during habituation and fixation duration—that is, shorter peak look associated with shorter fixation durations.
4. *Cognitive control*. An auditory stimulus anticipates a visual stimulus, which is presented repeatedly on one side for nine consecutive trials, before the target switches sides for the nine subsequent trials. Cognitive control is assessed as the proportion of correct anticipatory looks in the second, “postswitch” phase (Kovacs & Mehler, 2009). To our knowledge, no research has yet assessed the relationship of this measure to fixation durations during spontaneous orienting. However, given the role that inhibitory elements are thought to play in live eye movement control (Findlay & Walker, 1999a,b; Nuthmann et al., 2010), we predicted that we would find a negative relationship between fixation duration and cognitive control—that is, better cognitive control associated with shorter fixation durations. We also predicted that we would find a negative relationship between cognitive control and *intraindividual variance* in fixations—that is, greater cognitive control associated with reduced intraindividual variance in fixation durations.
5. *Arousal (indexed via tonic pupil size)*. Previous research from de Barbaro and colleagues has suggested a possible relationship between fixation duration and autonomic arousal. One measure of arousal used within infant cognition is pupil size, which primate work has demonstrated is robustly linked to activity within the norepinephrine (NE) system originating in the locus coeruleus, which has been associated with arousal/vigilance (larger pupil size = more arousal/alertness) (Aston-Jones & Cohen, 2005; Karatekin, 2007; see also Laeng et al., 2012)—although a range of other factors also influence pupil diameter, including luminance and higher-level factors such as cognitive load (e.g., Karatekin, 2007). Increased tonic pupil size (Anderson & Colombo, 2009), along with other indices of increased autonomic arousal (e.g., Van Engeland, 1984), has been noted in clinical conditions such as ASD, that also manifest reduced variability in fixation durations as well as shorter mean fixation durations (Benson et al., 2012; Kemner et al., 1998; Wass et al., under review1998). Therefore, we predicted that we would find a negative relationship with tonic pupil size—that is, increased tonic pupil size associated with shorter fixation durations, and with reduced intraindividual variance in fixation durations.

## Methods

### Participants

Twenty-one TD 11-month-old infants (12 male/9 female) participated in the study, which was spread across five separate laboratory visits over 15 (*SD* – 1.5) days. Infants were aged 335 (9.2) days at visit one. Different aspects of these data have been featured in previously published research (Wass, Porayska-Pomsta, & Johnson, 2011; Wass, Smith, & Johnson, 2012).

Participants completed two sets of assessments: Set A (free-viewing material) and Set B (experimental tasks).

#### Set A: Free-viewing material

Infants were seated on their caregiver's lap while the viewing material was presented. Stimuli were presented in Matlab on a 50 Hz Tobii 1750 eyetracker subtending 24° and the Talk2Tobii toolbox (Deligianni, Senju, Gergely, & Csibra, 2011). Shukla and colleagues verified the temporal latency of this toolbox using a similar hardware configuration and found it to be accurate to under 100 msec on more than 95% of samples (Shukla, Wen, White, & Aslin, 2011).

Free-viewing materials are shown in Figure 1. Five categories of stimulus (two static, three dynamic) were presented. Material in the categories “Simple shapes”, “Complex Static Scenes”, and “Multiple faces dynamic” and some of the material from “TV clips” were presented in a randomized order at visits 1 and 5 (c. 600 sec of material per session, presented in three different blocks); the remaining material was presented across visits 2, 3, and 4 (c. 830 sec of material per session). In addition, 64 sec of viewing material per participant was also presented of single faces (static and dynamic). However, this material proved unusable for fixation duration analysis because they contained a large number of saccades between close-by locations, which could not be identified accurately due to limitations in the accuracy of the eyetracker that we were using (see Wass et al. (2012) for a further discussion of this). Therefore, they have not been included in this analysis. All material was presented in segments of up to 135 sec in duration. Between segments, the experimenter decided whether to administer a break (if the infant had been fidgety or inattentive during the previous segment) or to proceed.

**Figure 2:**
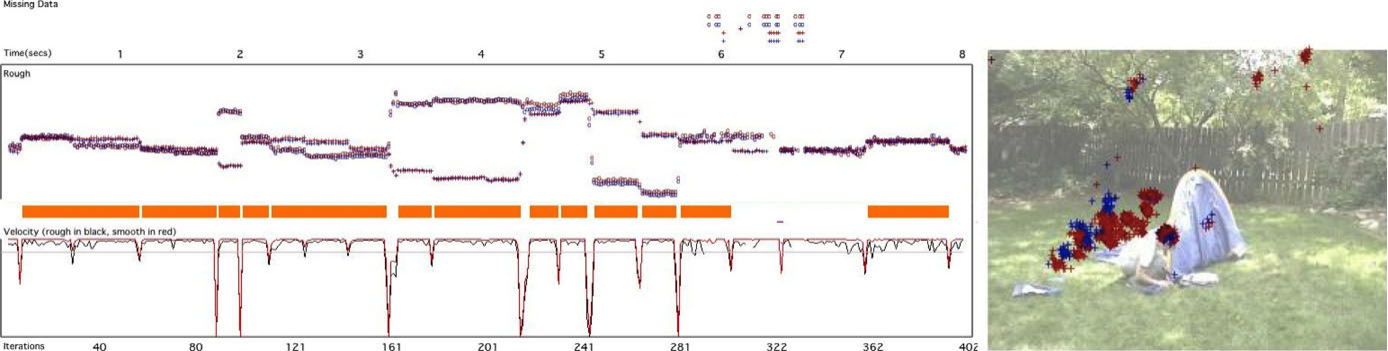
A sample of processed data showing the fixation parsing we conducted. This sample shows data from an infant viewing an 8-sec dynamic clip. Time is drawn on the *x*-axis. From top to bottom the plots show: missing Data; time (in sec); XY Gaze coordinates (X and Y both drawn on the *y*-axis); fixations detected by our algorithm (drawn as orange bars); velocity (i.e., rate of change in gaze position); time (in iterations). A screenshot of the dynamic clip the infant was viewing is drawn on the left, with the infant's gaze data superimposed in red and blue.

Fixation parsing was performed using Matlab scripts described in Wass et al., 2012 and available for download (http://www.mrc-cbu.cam.ac.uk/people/sam-wass/) (see Figure 2 and Supplementary Materials for more details).

#### Set B: Experimental tasks

The experimental tasks were conducted twice at visits 1 and 5 (at 15 days' interval). Each task was presented in blocks as described below; blocks were interleaved in an order that was pseudo-randomized (with the criterion that no two blocks of the same experiment were presented consecutively). This randomization was conducted to preclude the possibility of order effects being responsible for any observed relationships between tasks. Those viewing materials from Set A that were presented at visits 1 and 5 (as described above) were presented in three blocks, interleaved with the Set B measures.

1. *Psychomotor RTs*. The “gap-overlap” task was used to assess psychomotor RTs (noncompetition) and attentional disengagement latencies (Elsabbagh et al., 2008; Wass et al., 2011). After fixating a CT (a cartoon flower, subtending 4.5°), a lateral target (LT, a cartoon cloud, 3°) was presented to the left or right; when the participant fixated the LT, they received a brief audiovisual reward. In the noncompetition condition, the CT disappears concurrently with LT appearance; in the competition condition, the CT remains onscreen with LT appearance. Trials were presented in a pseudo-randomized order until enough valid trials had been collected (12 usable trials per condition per visit) or the infant became inattentive. The task was presented in two blocks; each block lasted approximately 2–3 min. The RT was the time elapsed between LT appearance and the reported position of gaze leaving the central fixation area (a 9° box around the CT). RTs for individual trials that were <200 or >1200 msec were excluded (following Elsabbagh et al., 2008). One participant who provided fewer than 10 valid trials per condition on each visit was excluded. “Psychomotor RT – noncompetition” was the mean RT in the noncompetition condition. “Psychomotor RT – disengagement” was the differential between mean RTs in the competition and noncompetition condition (following Elsabbagh et al., 2008).
2. *Processing speed* (*peak look during habituation)*. Four different still images were presented in two blocks of two at different stages of the testing protocol. Two images were of complex nonsocial scenes (e.g., butterflies on a field); two were of monochrome objects against a white background. Trials commenced once the participant fixated a CT and ended when they had looked away for 1 sec or more. Individual stimuli were presented repeatedly until two successive looks had taken place that were each <50% of the longest unbroken look so far, or until eight individual looks had elapsed. If a trial lasted more than 15 sec, a small (c. 0.4 deg) re-fixation target was briefly presented during the trial to confirm calibration validity; this did not affect the trial timing in any way. Our outcome measure was peak look—that is, the length of the longest trial (Colombo & Mitchell, 2009; Courage et al., 2006). Data from one participant were excluded as they did not complete both blocks at both pre- and post-testing. The duration of each block was infant controlled but typically lasted 1–3 min.
3. *Cognitive control task*. After fixating a CT (a cartoon flower subtending 4.5°), the trial commenced following a 300 msec delay. Two blank rectangles (11 × 9°) were presented left and right, concurrent with an auditory stimulus for 2000 msec (the anticipatory window). A visual reward (lasting 4000 msec) then appeared on one side (in either the left or right rectangle) for nine trials in a row (the preswitch phase) before swapping sides for the next nine trials (the postswitch phase). The task was presented in two blocks, each lasting 2–3 min. One participant did not complete all blocks across the two testing sessions and so has been excluded. If the participant correctly anticipated the presentation of the reward (defined as a saccade beginning between 300 and 2300 msec after trial onset and subject to a minimum look duration of 400 msec), then the visual reward stimulus appeared immediately. The measure was proportion of correct anticipatory looks between trials 11–18 (i.e., the postswitch phase) (following Kovacs & Mehler, 2009).
4. *Arousal* (*tonic pupil)*. The protocol used for tonic pupil size measurement was derived from Anderson and Colombo (2009). Tonic pupil was recorded during the administration of the gap-overlap task, as this was a task in which the cognitive load was minimal; color and luminance were also identical for all participants. A detailed description of the steps we took to ensure this, as well as the data parsing and analysis techniques that were used, can be found in the Data S1.

## Results

### Set A (free viewing) – Descriptive statistics

Before analyzing results for whether individual differences in fixation duration are stable across different stimulus types, we first performed descriptive analyses (see also Data S2). Table 1 compares the fixations observed to static versus dynamic stimuli. The number of fixations obtained is higher for the dynamic than for the static viewing materials, which reflects the fact that the total volume of viewing material was also higher (Figure 1). Intrasubject standard error is lower for dynamic than for static stimuli, which may also be due to the much larger number of fixations obtained for dynamic stimuli. A repeated-measures ANOVA was conducted to assess whether the differences between stimulus category in mean fixation duration reached significance. Mauchly's (1940) test was inspected and departure from sphericity was corrected using the Greenhouse-Geisser epsilon (Greenhouse & Geisser, 1959). Mean fixation duration was found to be significantly shorter for static than dynamic stimuli (*F*(1, 2.7) = 8.4, *p* = .001). This is consistent with previous reports from the adult literature (Dorr et al., 2010; Smith & Mital, 2013).

**TABLE 1:** Descriptive Statistics of Fixations Observed for the Different Categories of Visual Stimulus Presented in Set A

	*N* participants	Mean (msec)	Median (msec)	Avg intrasubject standard error	Avg intrasubject skewness	Avg intrasubject kurtosis	Intersubject standard error	Intersubject skewness	Intersubject kurtosis	Mean [std] *N* fixations
All static	20	590	474	28.37	22.20	3.24	16.93	−0.17	−1.00	323 (55)
All dynamic	21	755	540	20.55	80.67	6.37	20.49	0.44	−0.29	1637 (231)

#### How stable are interindividual differences in fixation duration?

Our first research question was, are fixation durations during the viewing of complex, naturalistic scenes a stable parameter of individual differences during infancy? We addressed this question in two ways.

**Figure 3:**
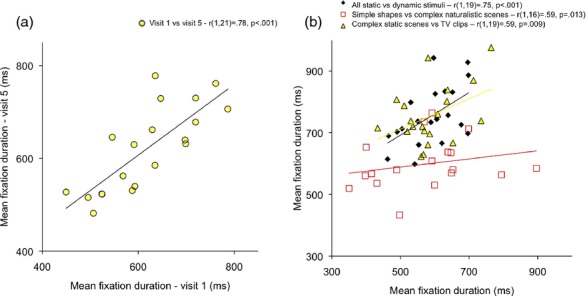
Stability of interindividual differences: (a) across testing sessions. An identical battery of mixed static and dynamic viewing material was presented twice at 15 days' interval (visits 1 and 5). (b) across different stimulus types. Data were pooled across all five visits, and a comparison of mean fixation duration across different types of stimulus was conducted. The legends show Pearson's product moment correlations as reported in the main text and Table 2.

First, we looked at test–retest reliability when an identical battery of mixed static and dynamic viewing material was administered twice to the same participants at 15 days' viewing interval (on visits 1 and 5). A Kolmogorov–Smirnov (KS) test showed that both sets of results were normally distributed (*p *≥ .2), and so parametric analyses have been reported. The Pearson's product moment correlation between visit 1 and visit 5 was *r*(16) = .78, *p*(two-tailed) <.001 (see Figure 3a). This relationship was unaffected by calculating a partial correlation between visits 1 and 5 that includes the difference in data quality between the two visits as a covariate (*r*(13) = .82, *p* < .001). This suggests that the observed correlation is not attributable to stable individual differences in data quality obtained across the two visits. Other measures also showed test–retest reliability between the two visits: intrasubject variance in fixation duration (*r*(18) = .55, *p* = .01) and intrasubject kurtosis (*r*(18)  = .53, *p* = .02). Intrasubject skewness was marginally not significant (*r*(18) = .39, *p* = .09).

Second, we looked at the stability of interindividual differences in mean fixation duration across the different types of visual stimulus we presented, averaging across visits. The relationship between mean fixation duration for all static and mean fixation for all dynamic stimuli was *r*(18) = .60, *p* = 0.007. Additionally, Table 2 shows a breakdown including the two different subcategories of static stimulus and three subcategories of dynamic stimulus (as described in Figure 1). KS tests suggested that all results were normally distributed (*p *≥ .05) so parametric analyses have been reported. Pearson's product moment correlations, together with the number of fixations available in each stimulus category, are shown in Table 2. Medium positive correlations were noted across all comparisons, although not all reached significance.

**TABLE 2:** Correlations of Mean Fixation Duration (in msec) for the Different Stimulus Types We Administered

	Complex scenes static: 220 (32)	Multiple faces dynamic: 74 (6)	TV clips: 1206 (171)	Naturalistic scenes: 410 (68)
*Simple shapes static (N: 97 [26])*	0.24	0.56**	0.39(*)	0.64**
*Complex scenes static (N: 220 [32])*		0.44*	0.43*	0.17
*Multiple faces dynamic (N: 74 [6])*			0.55**	0.47**
*TV clips (N: 1206 [171])*				0.64**

** Correlation is significant at *p* < 0.01

* *p* < 0.05,

(*) *p* < 0.1. All degrees of freedom = 19. The figures after each category name show the average and the standard error of the number of fixations available per participant in each category.

#### Relationship of fixation duration to experimental assessments of infant attention

Our second research question was, how do individual differences in fixation duration relate to performance on other experimental assessments of attention?

### Set B (experimental tasks) – Descriptive statistics

Prior to addressing this question we first performed descriptive analyses on our Set B data; we also calculated the test–retest reliability of our measures, using identical calculations to those used for the fixation duration measure above (see Table 3). Significant correlations between visit 1 and visit 5 were observed for psychomotor RT – disengagement (*p* < .05), peak look duration (*p* < .001), cognitive control (*p* < .01) and tonic pupil size (*p* < .001). The test–retest reliability of psychomotor RT – noncompetition approached but did not reach significance (*p* = .09). We also analyzed the relationships between the different tasks administered within set B and identified no significant relationships between measures (see also Table 3).

**TABLE 3:** Descriptive Analyses for the Set B Measures

Descriptives	Mean	Standard error	*N* participants	Test–retest reliability^a^
Psychomotor RT (noncompetition) (msec)	349	27	20	.38 (.09)
Psychomotor RT (disengagement) (msec)	270	63	20	.51 (.03)
Processing speed (peak look during habituation) (sec)	36.3	18.7	20	.75 (<.001)
Cognitive control (prop. corr.)	0.32	0.17	19	.60 (.003)
Arousal (tonic pupil) (millimeter)	4.32	0.71	20	.94 (<.001)

Correlations between measures^b^	Psychomotor RT (disengagement) (millisecond)	Processing speed (peak look during habituation) (sec)	Cognitive control (prop. corr.)	Arousal (tonic pupil) (millimeter)
Psychomotor RT (noncompetition) (msec)	0.12	−0.24	−0.25	0.28
Psychomotor RT (disengagement) (msec)		0.30	−0.02	0.15
Processing speed (peak look during habituation) (sec)			0.21	0.04
Cognitive control (prop. corr.)				0.38

note

RT = reaction times.

a Pearson's product moment correlation co-efficients (*p* values in bracket) showing test–retest reliability when an identical assessment battery was administered twice at 15 days' interval – see description in text.

b Correlations in performance between measures. All values shown are the Pearson's product moment correlations. No significant relationships were identified (all *p* values >0.1).

### Relationship between Set A and Set B

Table 4 shows the results of analyses we conducted to examine this question. KS tests suggested that all variables were normally distributed (*p* > 0.08), and so parametric statistics have been reported.

**TABLE 4:** Zero-order Correlations and Multiple Regression Calculations to Examine the Relationship Between Fixation Duration to Dynamic and Static Stimuli and Our Experimental Assessments of Attention

	Psychomotor RT (noncompetition)	Psychomotor RT (disengagement)	Processing speed (peak look during habituation)	Cognitive control	Arousal (tonic pupil)
All dynamic – fixation duration (*M*)					
Zero-order correlation					
*r*	0.50	0.27	−0.17	−0.15	−0.17
*p*	.03*	.26	.48	.55	.47
Multiple regression					
*b*	1.96	0.32	0.00	0.11	−0.05
SE	0.74	0.32	0.00	0.12	0.03
*t*	2.65	1.01	−0.80	0.88	−1.70
Part correlations	0.54	0.21	−0.16	−0.18	−0.35
*p*	.02*	.33	.44	.4	.12
All static – fixation duration (*M*)					
Zero-order correlation					
*r*	0.25	0.19	−0.17	−0.51	−0.49
*p*	.31	.43	.5	.03*	.03*
Multiple regression					
*b*	0.67	0.33	0.00	−0.11	−0.05
SE	0.73	0.29	0.01	0.11	0.03
*t*	0.93	1.14	−0.53	−0.92	−2.00
Part correlations	0.19	0.24	−0.11	−0.19	−0.42
*p*	.37	.28	.61	.38	.07(*)
All dynamic – fixation duration (within-participant variance)					
Zero-order correlation					
*r*	0.13	−0.10	−0.34	−0.67	−0.49
*p*	.58	.69	.14	.01*	.03*
Multiple regression					
*b*	0.01	0.01	0.00	−0.02	0
SE	0.04	0.02	0.00	0.01	0.00
*t*	0.22	−0.43	−1.4	−2.4	−1.16
Part correlations	0.04	−0.08	−0.25	−0.43	−0.21
*p*	.83	.67	.19	.03*	.27
All static – fixation duration (within-participant variance)					
Zero-order correlation					
*r*	0.25	−0.08	−0.43	−0.50	−0.39
*p*	.59	.64	.07(*)	.03*	.09(*)
Multiple regression					
*b*	−0.01	0.02	0.00	−0.02	−0.01
SE	0.12	0.05	0.00	0.02	0.01
*t*	−0.07	0.45	−1.34	−1.24	−1.24
Part correlations	−0.02	0.10	−0.29	−0.27	−0.27
*p*	.94	.66	.21	.24	.24

Note

RT = reaction times.

For each variable, the Pearson's correlation coefficient and two-tailed *p* value of the zero-order correlation have been given. For the multiple regression, the unstandardized regression coefficient has been given along with its standard error. The *t*-test value of the regression coefficient is also shown along with the part correlation.

We conducted our analyses using four separate dependent variables: (1) mean fixation duration to dynamic stimuli, (2) mean fixation to static stimuli, (3) within-participant variance (Standard Error of the Mean – *SEM*) in fixations to dynamic stimuli, (4) within-participant variance (*SEM*) in fixations to static stimuli.

For each dependent variable, we first examined zero-order correlations with the Set A measures. We then used multiple regression to examine the unique relation of each of the Set A measures to our dependent variables. In these analyses, all Set A measures were entered simultaneously. The ratio of participants to predictors was ranged from 3.8 to 4.2; it should be noted that this is below the prescribed ratio of five suggested by Hair and colleagues, which means that there is a risk of over fitting (Hair, Tatham, Anderson, & Black, 1998; but see Arrindell & van der Ende, 1985; Field, 2013). Overall, the fit of the model was marginally nonsignificant *F*(5,15) = 2.70, *p* = .07, which may reflect this and means that results should be treated with caution. The model accounted for ∼60% of the variance (*R*^2^ = .68, adjusted *R*^2^ = .60).

For the sake of clarity our discussion of the results is organized into four sections, based on our initial predictions:

1. *Prediction (a)* – *a positive relationship with psychomotor RTs—that is, faster RTs associated with shorter fixation durations*.

Zero-order correlations suggested that psychomotor RT (noncompetition) correlated significantly with mean fixation duration to dynamic stimuli (*r* = .50, *p* = .02) but not to static stimuli (*r* = .25, *p* = .31). These findings were relatively unchanged in the multiple regression model: The part correlation between fixation duration to dynamic stimuli and psychomotor RT was .54, compared with a zero-order correlation of .50. This suggests that noncompetition psychomotor RTs explain a largely discrete proportion of the variance in fixation duration to dynamic stimuli.

Second, we looked at how noncompetition psychomotor RTs relate to average within-participant variance in fixation duration. In contrast to the mean measure, we found no relationships between psychomotor RT and within-participant variance in fixation duration to either dynamic stimuli (zero-order correlation: .13, part correlation: .13) or to static stimuli (zero-order correlation: .25, part correlation: −.02).

Next, we conducted identical analyses to examine the relationship between our fixation duration measures and psychomotor RT (disengagement). Here, we found no significant relationships between attentional disengagement and fixation duration to either dynamic (*r* = .27) or static (*r* = .19) stimuli. Attentional disengagement also showed no significant relationship with within-participant variance in fixation duration for either dynamic (*r* = −.10) or static (*r* = −.08) stimuli.

1. *Prediction (b)* – *a positive relationship with processing speed (indexed via peak look during habituation)—that is, shorter peak look associated with shorter fixation durations*.

Peak look during habituation did not relate significantly to fixation durations during the viewing of either dynamic (*r* = −.17) or static stimuli (*r* = −.17). No significant relationships were identified between peak look during habituation and intraindividual variance in fixation duration during the viewing of either dynamic (*r* = −.34) or dynamic (*r* = −.43) stimuli.

1. *Prediction (c)* – *a negative relationship with cognitive control—that is, better switching associated with shorter fixation durations*.

For cognitive control, the opposite pattern was observed to that found for the psychomotor RT measure: A significant relationship was observed with fixations to static stimuli (*r* = −.51, *p* = .02) (increased performance on switching task associated with shorter fixation duration), but no relationship was observed with fixations to dynamic stimuli (*r* = −.15, *p* = 53). However, the relationship between mean fixation duration to static stimuli and cognitive control was found to be weaker in the multiple regression model (part. corr. = −.19), suggesting that the proportion of the variance in fixation duration accounted for by cognitive control was accounted for by our other independent variables.

For intraindividual variance in fixation duration, consistently negative correlations were identified (i.e., increased performance on switching task associated with reduced variance in fixation duration). This was observed independently for fixations to static (*r* = −.50, *p* = .03) and to dynamic (*r* = −.67, *p* = .01) stimuli. Again these relationships were found to be weaker in the multiple regression model, although the relationship between cognitive control and within-participant variance in fixation duration to dynamic stimuli was still found to be significant (part correlations – *r* = −.27 for static and *r* = −.43 for dynamic).

1. *Prediction (d) a negative relationship with arousal (indexed via tonic pupil size)— that is, increased tonic pupil associated with shorter fixation durations*.

A similar pattern of results was observed for the arousal measure as for the switching measure: a relationship with fixation duration to static (*r* = −.49, *p* = .03) but not dynamic (*r* = −.17, *p* = .47) stimuli. The relationship between arousal and mean fixation duration to static stimuli was only marginally weaker in the multiple regression model (part correlation −.42), although it became nonsignificant. This suggests that tonic pupil explains largely but not entirely discrete proportions of the variance in fixation duration to static stimuli.

Consistent relationships were observed between arousal and within-participant variance in fixation duration to static (*r* = −.49, *p* = .03) and dynamic stimuli (*r* = −.39, *p* = .09). However, these relationships became weaker in the multiple regression model (part correlations: static: −.27, dynamic: −.21), suggesting that the proportion of the variance they explain is shared across other independent variables.

## Discussion

This study addressed two questions. Our first question was, can stable individual differences be identified in fixation durations during the viewing of complex naturalistic scenes in infancy? We addressed this question in two ways. First, we presented an identical mixed static/dynamic viewing battery to TD 11-month-old infants at 15 days' interval and found significant test–retest reliability on mean fixation duration. Second, we found that interindividual differences in fixation duration remained stable across the different types of viewing material (Table 2 and Figure 3) despite the differences we found in fixation patterns to different types of visual stimulus. This replicates similar reports from the adult (Andrews & Coppola, 1999; Castelhano & Henderson, 2007) and nonhuman primate (Kano & Tomonaga, 2011a) literature. The relationship we observed was unaffected by covarying for data quality, suggesting that these findings are probably not attributable to methodological factors associated with fixation parsing (Wass et al., 2012). Mean fixation duration was found to be significantly shorter for static than for dynamic stimuli, which replicates previous findings with adults (Dorr et al., 2010; Smith & Mital, 2013). This may be because the total amount of information content at each fixation point is higher for dynamic than for static stimuli, or because it is harder to disengage from a moving than a static fixation location.

Our second question was, how do individual differences in spontaneous fixation behavior during unconstrained orienting relate to performance on other experimental assessments of infant attention? Given the small sample size of the present study, our findings here must be interpreted with caution; nevertheless, a few observations can be drawn. For most measures, we observed effects that were in line with our predictions; we also found directional effects that were consistent across static and dynamic stimuli (Table 4). Although the fit of the multiple regression model was marginally nonsignificant (*p* = .07) meaning that results should be treated with caution, the results of the zero-order correlations were largely corroborated by the multiple regression analysis; this suggests that, as predicted, our experimental attention assessments were explaining largely discrete proportions of the variance in fixation duration (with the exception of cognitive control and arousal (tonic pupil size) which we discuss below).

We found independently for fixations to both static and dynamic stimuli that within-participant variance in fixation duration appeared most strongly related to cognitive control and arousal (indexed via tonic pupil size). Increased cognitive control and increased arousal were associated with reduced variability in fixation duration. These results are consistent with the literature on arousal and fixation durations within clinical populations such as ASD, where shorter fixation durations and reduced variability in fixation duration have been noted (Benson et al., 2012; Kemner et al., 1998; Wass et al., under review1998), together with larger tonic pupil size (Anderson & Colombo, 2009) and relatively spared cognitive control (Geurts, Corbett, & Solomon, 2009). They may, however, be inconsistent with Bronson (1994) who looked at the relationship between change in pupil size during a testing session and changes in the proportion of time spent in brief fixations, although exactly how Bronson defined his measures is unclear (Bronson, 1994, p. 1256).

Our results replicate those of de Barbaro and colleagues in suggesting the importance of general vigilance levels in mediating micro-temporal orienting behaviors during infancy (de Barbaro et al., 2011). The arousal/alertness component of attention is thought to involve the NE system originating in the locus coeruleus (Aston-Jones & Cohen, 2005; Aston-Jones et al., 1999, 2007). Primate work has demonstrated a robust relationship between activity levels within this system and tonic pupil size (larger pupil size = more arousal/alertness) (Aston-Jones & Cohen, 2005; Karatekin, 2007; see also Anderson & Colombo, 2009), and a link with cognitive control during infancy has been postulated (e.g., Rothbart, Ellis, Rueda, & Posner, 2003; Sheese, Rothbart, Posner, White, & Fraundorf, 2008). The NE system is thought to mediate shifting between different modes of attention (e.g., “selective” versus “scanning” attention) (see e.g., Aston-Jones et al., 1999; Pannasch, Helmert, Roth, Herbold, & Walter, 2008). In individuals with high tonic arousal, this is thought to become aberrant (Aston-Jones et al., 2007), leading to fixational eye movement patterns that are more invariant and less subject to short-term variability due to changes in the target element—a pattern that has been reported, for example, in hyperaroused individuals with ASD (Anderson & Colombo, 2009; Benson et al., 2009, 2012). However, more work—demonstrating, for example, that phasic increases in arousal correlate with phases of reduced variance in fixation duration—is necessary to understand these changes in more detail.

Although all directional effects were observed consistently across static and dynamic stimuli and were broadly in line with our predictions, we also found that some measures showed markedly stronger relationships for dynamic than for static scenes, and *vice versa*. Noncompetition psychomotor RT related to mean fixation duration during dynamic stimulus viewing (*r* = .50), which remained significant even when the other experimental variables were included in the regression model, but the same measure showed only a weaker, not significant relationship to mean fixation duration during static stimulus viewing (*r* = .25). For cognitive control and arousal we found the opposite relationship: both measures showed weak, nonsignificant relationships with fixation duration to dynamic stimuli (*r* = −.15/−.17 for cognitive control/arousal) but significant relationships with fixation duration to static stimuli (*r* = −.51/−.49).

Although these relationships require replication, nevertheless a tentative *post hoc* interpretation can be attempted. Dynamic scene viewing involves a greater proportion of reactive saccades that are triggered in response to saliency changes within the target element (Dorr et al., 2010; Itti & Baldi, 2009; Itti & Koch, 2001; Mital, Smith, Hill, & Henderson, 2010). During static scene viewing, in contrast, there is no attentional capture due to changes in the target element (Henderson et al., 2009). During static stimulus viewing, therefore, a greater proportion of saccades are thought to be internally generated, influenced by internal stochastic saccade timer mechanisms (Henderson & Smith, 2009) or other endogenous features (see also Findlay & Walker, 1999a,b; Nuthmann et al., 2010).

Whatever their interpretation, our findings are striking given that the vast majority of research into individual differences in infant attention has, for practical reasons, used static stimuli. Research in this area may in future help us understand the findings reported on how spontaneous orienting differs in atypical development (conditions such as ASD and Attention Deficit Hyperactivity Disorder), some of which have used static and others more naturalistic, dynamic stimuli (e.g., Chawarska, Volkmar, & Klin, 2010; Jones, Carr, & Klin, 2008; Speer, Cook, McMahon, & Clark, 2007).

There were two further areas where our results were not as predicted. The first was the relationship between processing speed (indexed via peak look duration during habituation) and fixation duration. Here, instead of a positive relationship, we observed nonsignificant weak negative correlations (*r* = −.17/−.17 for dynamic/static). Second, of the two psychomotor RT measures we included, we found that the noncompetition psychomotor RT task, (i.e., the noncompetition condition) related to fixation durations (*r* = .50/.25 for dynamic/static) more strongly than the disengagement measure (the differential between the competition and noncompetition conditions) (*r* = .27/.19 for dynamic/static). This finding can be contrasted to the literature on patterns of “sticky fixation” behavior observed on gross orienting behaviors—looks to versus away from a static picture of a face of checkerboard—in younger infants, where Frick et al. (1999) found, in TD 3- to 4-month-old infants, that longer-looking infants were slower to shift fixation to peripheral stimuli on the competition trials but not on the noncompetition trials. However, it should be remembered that infants in the present study were 11-month-olds and that the literature on “sticky fixation” issues generally concerns infants earlier during the first year of life (Atkinson, 2000; Atkinson & Braddick, 1985, 2012; Hood & Atkinson, 1993; Hood, 1995). Therefore, a different pattern may be observed if our experiment is repeated with younger infants.

Although the infant neuroimaging data in this field are equivocal and limited (Braddick et al., 1992; Csibra et al., 1998; Csibra, Tucker, & Johnson, 2001), research from the adult and primate literature suggests that disengaging visual attention involves additional cortical involvement relative to attention shifting under noncompetition conditions (Findlay & Walker, 1999a,b; Nuthmann et al., 2010). Alternatively, therefore, our finding that psychomotor RT latencies under noncompetition conditions were more predictive of fixation duration may be consistent with other authors who speculated that subcortical processing systems may play a larger role in mediating attentional orienting during the first year of life (Colombo & Cheatham, 2006; Johnson, 1991; Posner, Rothbart, Sheese, & Voelker, 2012; Schiller, 1985). This model may make contrasting predictions to the “sticky fixation” model described above. Whereas the “sticky fixation” model might predict a relationship between fixation duration and disengagement latencies early during the first year, while poor attentional disengagement limits orienting behavior, but not later in development, the “increasing cortical control” model predicts that the relationship might *increase* with increasing age, as cortical control over fixational eye movement behavior increases. In future, longitudinal studies (cf. Hunnius et al., 2006) as well as studies with co-registered EEG will help us to understand these developmental changes in more detail.

## Limitations and Future Plans

First, the low sample size of the present study has precluded factor analyses (cf. e.g., Rose, Feldman, & Jankowski, 2004) and means that the results of the multiple regression analysis should be treated with caution. Second, these results require replication. Third, as with any correlational findings, conclusions as to causal interactions must be treated with caution. Better ways of assessing causal interactions include the use of structural equation modeling (Rose et al., 2012) and targeted cognitive training (Wass et al., 2011).

Fourth, we have pooled together all fixation durations recorded during the presentation of particular stimuli, irrespective of the spatial location of the fixation. This is because *post hoc* verification checks found that the disparity between infants' actual position of gaze and that reported by the eyetracker was in many cases >1° (Bronson, 1990, 1994). Unfortunately, the accuracy of gaze position was not validated on a per participant basis (cf. Frank, Vul, & Saxe, 2012). In future, with the addition of better *post hoc* calibration checks, it will be possible to analyze the relationship of fixation duration to the information content at the point fixated—either using dynamic areas of interest (e.g., Frintrop, Rome, & Christensen, 2012) or using algorithms that break down a dynamic image frame-by-frame into constituent properties including corners and edge orientations, flicker and motion (e.g. Mital et al., 2010). Using these methods it will be possible to assess whether there are three individual differences in the degree to which fixation durations are influenced by exogenous, stimulus-driven factors versus endogenous factors (such as the semantic content at the point fixated – e.g. face versus nonface) (cf. Berg, Boehnke, Marino, Munoz, & Itti, 2009; Frank, Vul, & Johnson, 2009; Itti & Baldi, 2009; Mital et al., 2010). Fifth, a further factor to investigate is the different role that audio plays in influencing eye movements. The stimuli used in the present experiment contained a mix of diegetic and nondiegetic audio.

Sixth, we have only examined fixation durations while naturalistic scenes were presented 2-D on a computer screen. A future goal is to apply the methods used here to data from a head-mounted eyetracker (Aslin, 2009; Corbetta, Guan, & Williams, 2011; Franchak & Adolph, 2011; Franchak, Kretch, Soska, & Adolph, 2011). This will allow us to investigate in more detail how individual differences emerge in early naturalistic gaze behavior during both typical and atypical development, and how these differences relate to other long-term parameters of cognitive development.

## Summary

These results are one of the first attempts to identify and to understand the factors guiding individual differences in infant oculomotor behavior during the viewing of complex naturalistic scenes. First, we identified evidence that individual differences in fixation durations are stable across time (at 15 days' interval) and across different types of viewing material. Second, we found evidence that, during the viewing of both static and dynamic stimuli, variance in fixation duration was significantly related to both cognitive control and arousal (indexed via tonic pupil size) (increased cognitive control and increased arousal associated with reduced variability in fixation duration). Third, we found that mean fixation durations during the viewing of dynamic viewing material related most strongly to psychomotor RT measures, but that fixation durations during the viewing of static viewing material related most strongly to cognitive control and arousal.

These results open a number of avenues for further work. First, the differential pattern of individual differences we found between viewing behavior to dynamic versus static stimuli requires replication—Particularly, given that the majority of work with both typical and atypical populations hitherto has used static stimuli. Second, we have discussed how future work will allow us to move beyond studying mean fixation duration using the relatively crude techniques we have presented here, and toward quantifying individual differences in the ability to modulate microtemporal orienting behaviors contingent on context, as a result of exogenous and endogenously relevant aspects of information content at the point fixated. This work therefore offers new potentials for exploring and quantifying individual differences in naturalistic orienting behavior.

## Footnotes

Within the infant literature it should be noted that there are also researchers who use the term “fixation” to refer to looks to a screen or stimulus presentation area (see e.g., Richards & Cronise, 2000), rather than in the more technical usage within the adult oculomotor literature that we adopt here, defined as the period between saccadic eye movements during which the eyes are relatively stable and gaze velocity is low (Holmqvist et al., 2011).

## Supporting Information

Data S1 Supplementary Methods: Further details of fixation duration parsing. Further details of tonic pupil parsing. **Data S2.** Supplementary Results: Fixation durations - comparison with previously reported infant and adult fixation durations. **Figure S1**. Sample raw data-plots of tonic pupil data. **Figure S2.** Scatterplot showing the zero-order correlations reported in Table 4.
