# Supplementary material for: Individual Differences in Infant Oculomotor Behavior During the Viewing of Complex Naturalistic Scenes
Source: Infancy. 2014 Mar 28;19(4):352–84. doi: 10.1111/infa.12049 (PMC4286103; doi:10.1111/infa.12049)
Supplement: Data S1 — Supplementary Methods: Further details of fixation duration parsing. Further details of tonic pupil parsing. Data S2. Supplementary Results: Fixation durations - comparison with previously reported infant and adult fixation durations. Figure S1. Sample raw data-plots of tonic pupil data. Figure S2. Scatterplot showing the zero-order correlations reported in Table 4. [file infa0019-0352-SD1.docx]

**Supplementary Methods**

Details of fixation duration parsing

Briefly, the analysis procedure was as following: 1) smoothing was performed using a bilateral filtering algorithm written by Ed Vul (Frank, Vul, & Johnson, 2009; based on Durand & Dorsey, 2002). 2) interpolation was performed (based on the average x- and y-coordinates since the start of the fixation) to cover periods of data loss up to 150ms. 3) velocity thresholding was performed using a velocity threshold of 35˚/sec (see e.g. Holmqvist et al., 2011). 4) false (artifactual) fixations and saccades) were identified based on the following criteria: a) fixation is a complete fixation; b) displacement since previous fixation is >0.25˚; c) average velocity during previous fixation is < 12˚/sec; d) velocity in the three samples immediately preceding the saccade is < 12˚/sec; e) binocular disparity is not above 3.6˚; the fixation identified has a minimum temporal duration of 100ms. The rationale and validation procedures behind these processing steps are outlined in detail in Wass, Smith & Johnson (2012). One limitation of these algorithms is that they are not able to distinguish sections of smooth pursuit. Techniques used by adult researchers to identify smooth pursuit (e.g. Berg, et al., 2009; Nyström & Holmqvist, 2010) were unamenable to the lower-quality and lower temporal resolution eyetracker data obtained from infants. This point is discussed further below.

Wass, Smith & Johnson (2012) also contains analyses investigating how the eyetracker data quality obtained from different individuals relates to the fixation durations returned by our algorithms (cf. Aslin, 2012; Shic et al., 2009). For the present paper we repeated these analyses in order to replicate our findings. In contrast to the analyses previously reported, analyses on the current dataset suggested that the results of fixation parsing applied to individuals who show particularly low reliability in reported position of gaze (defined as a within-fixation variance of >0.5° using the metrics from Wass, Smith & Johnson (2012)) are still significantly influenced by individual differences in data quality; therefore data from these testing sessions has been removed from the current data set. This has led to results from one participant being excluded completely, and partially (on a session-by-session basis) from a further 6 participants. These exclusions do not affect the overall significance of any of the analyses reported in this paper.

*Tonic pupil*

The procedure we used for calculating tonic pupil size was based on Anderson & Colombo (2009; see also Chatham et al., 2009; Gredeback et al., 2012; Jackson & Sirois, 2009; Smallwood et al., 2011). We recorded tonic pupil size during presentation of the gap-overlap task (see Methods section). This was a task with minimum cognitive load (e.g. no memory or cognitive control requirements), and during which the viewing materials were closely matched across all participants. During this task, a uniformly coloured background was presented (subtending 24˚). Central and lateral targets consisting of green, grey, brown or yellow coloured cartoon animals (subtending 3˚-4.5˚) were presented individually as described in the main text. A consistent number of trials from each of three conditions (gap, baseline, overlap) was presented to all infants and stimuli were identical for all participants. Lateral stimuli were counterbalanced between left and right. (This means that reports of error in pupil size measurement from Tobii eyetrackers on deviation from the midline are not telling in the present instance, since errors are reported to be zero sum around the x dimension (Brisson et al., in press)). The task was presented in 4 blocks at different stages of the testing protocol and across visits 1 and 5. Each block lasted approximately 2-3 minutes. The same testing set-up (monitor, distance from monitor etc) was used for all participants and the lighting conditions and screen settings were kept exactly constant throughout.

Data were recorded at 50Hz; separate values for pupil dilation were obtained for the left and right eye using in-built algorithms supplied by the eyetracker manufacturer. Visualisations of the raw data (see Figure S2) suggested that the data showed different types of error: occasional egregious outliers, varying degrees of sampling error from one iteration to the next (manifesting as 'high-frequency noise' - see sample b in Figure S2), together with varying degrees of lost data contact. Other groups have used interpolation algorithms for tonic pupil estimations (eg Anderson & Colombo, 2009); however it was judged that a simple average was likely to be more accurate. The median was calculated rather than the mean because of problems noted with occasional egregious outlying values. The number of valid samples available varied between participants from 5454 to 7258 samples; median values were calculated independently then averaged for the left and right eyes.


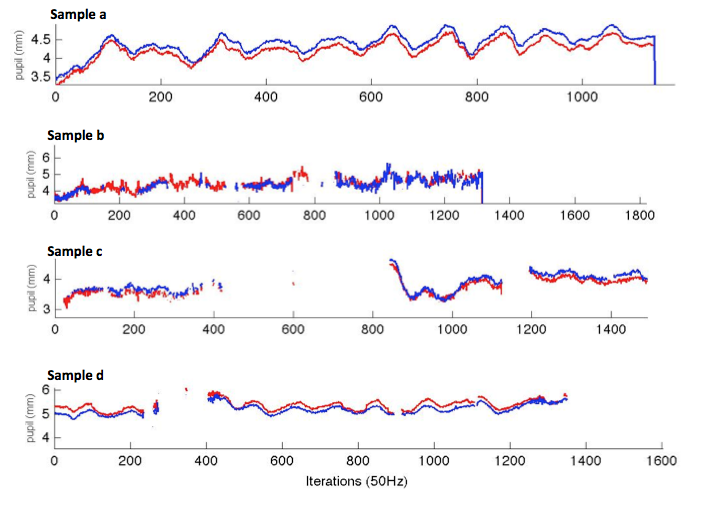


*Figure S1: Sample raw data-plots of tonic pupil data. Samples of pupil size data from four different infants are shown, that were recorded during administration of the gap-overlap task. For each individual data from the left eye are drawn in red and those from the right eye are drawn in blue.*

**Supplementary Results**

*Fixation durations - comparison with previously reported infant and adult fixation durations*. As reported previously, we found mean fixation durations in the range 550-800ms for different types of visual stimulus (Wass, Smith & Johnson, 2012). These figures are higher than the 300-400ms traditionally reported in the adult literature (e.g. Dorr et al, 2010); although comparable to the median values of circa 600ms reported for 6-26-week-olds viewing static stimuli by Hunnius & Geuze (2004a) they are longer than the 403ms reported for 1-year-olds viewing dynamic stimuli by Kirkorian et al. (2012). There are a number of reasons for this discrepancy. First we have argued elsewhere that many of the fixation detection algorithms traditionally used to parse infant eyetracker data return fixations that are incomplete or fragmentary; the algorithms used here include several *post hoc* verification procedures to ensure that only complete fixations are recorded, which leads to a substantial increase in the mean values (Wass, Smith & Johnson, 2012). Second, and in contrast to other authors, we have also included very long (>1200ms) fixations in our means (e.g. Castelhano & Henderson, 2007; Nuthmann et al., 2010). Third, we have no criteria for rejecting smooth pursuit. However we found that when typically developing 11-month-olds view static viewing material (for which there is no possibility of smooth pursuit), 4.8% of the fixations we identified were longer than 1200ms, and 1.3% were longer than 2000ms, suggesting that differences are not limited to smooth pursuit. (For comparison, dynamic stimuli showed 11.5% of fixations longer than 1200ms and 4.2% longer than 2000ms.) Work with adults using algorithms that identify separate smooth pursuit segments has still found markedly longer fixation durations for dynamic than for static stimuli (Dorr et al., 2010; Smith & Mital, 2013).

*
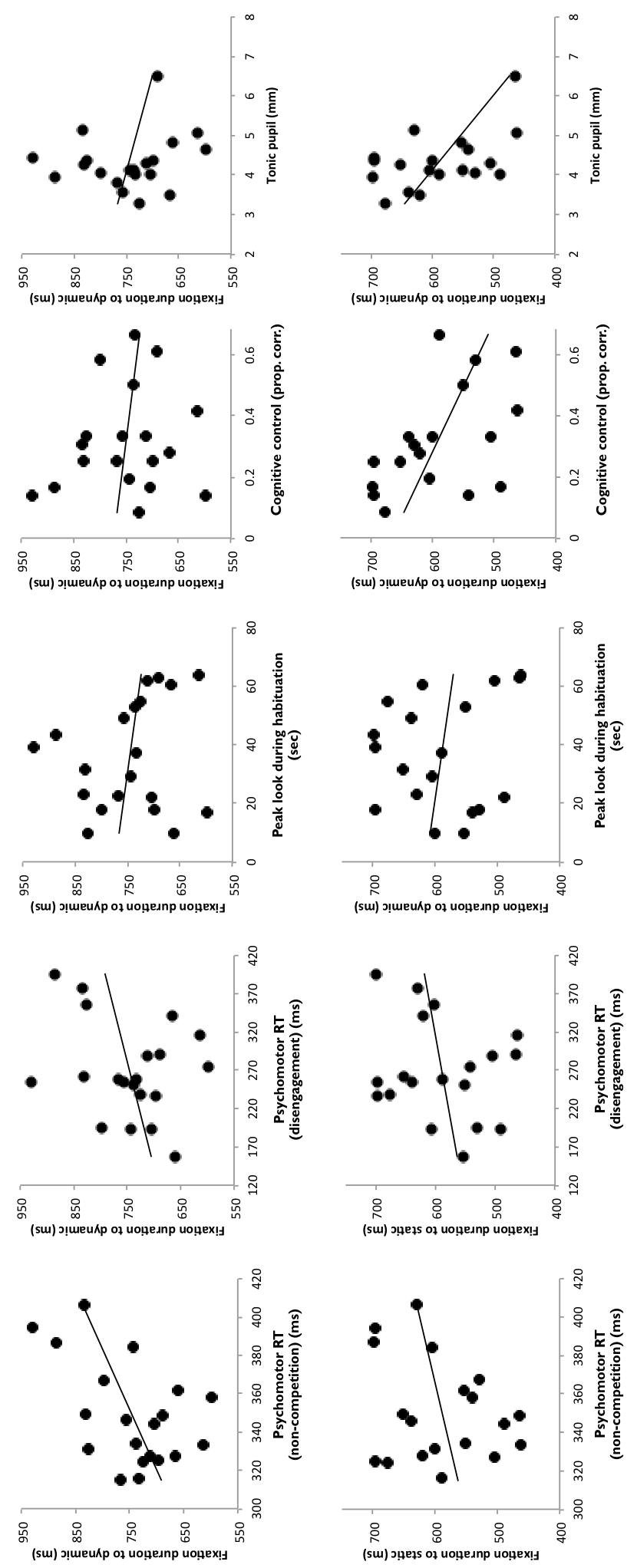
*

*Figure S2 – Scatterplot showing the zero-order correlations reported in Table 4. Only correlations for mean fixation duration are shown.*

**Supplementary References**

Chatham, C. H., Frank, M. J. & Munakata, Y. (2009) Pupillometric and behavioral markers of a developmental shift in the temporal dynamics of cognitive control. Proceedings of the National Academy of Sciences of the United States of America 106(14): 5529-5533.

Gredeback, G., Johnson, S. & von Hofsten, C. (2010). Eye Tracking in Infancy Research. Developmental Neuropsychology 35(1): 1-19.

Jackson, I. & Sirois, S. (2009) Infant cognition: going full factorial with pupil dilation. Developmental Science 12(4): 670-679.

Kirkorian, H. L., Anderson, D. R. & Keen, R. (2012). Age Differences in Online Processing of Video: An Eye Movement Study. Child Development 83(2): 497-507.

Smallwood, J., Brown, K. S., Tipper, C., Giesbrecht, B., Franklin, M. S., Mrazek, M. Carlson, J. M. & Schooler, J. W. (2011). Pupillometric Evidence for the Decoupling of Attention from Perceptual Input during Offline Thought. PLoS ONE 6(3).
